# A Case of Tacrolimus Maculopathy

**DOI:** 10.3390/clinpract12030033

**Published:** 2022-05-01

**Authors:** Martina Santarelli, Marco Zeppieri, Carlo Salati

**Affiliations:** Department of Ophthalmology, University Hospital of Udine, 33100 Udine, Italy; martina.santarelli21@gmail.com (M.S.); carlo.salati@asufc.sanita.fvg.it (C.S.)

**Keywords:** tacrolimus, transplantation, maculopathy, ocular toxicity, outer nuclear layer

## Abstract

(1) Background: Tacrolimus is an immunosuppressive agent commonly used in the management of solid organ allogeneic transplants in the prevention of rejection. Serious ophthalmic adverse effects with Tacrolimus have been reported in the literature, which includes cortical blindness and optic neuropathy. (2) Methods: We describe a rare case of maculopathy as a possible complication of Tacrolimus therapy. A 56-year-old man receiving Tacrolimus for immunosuppression after liver transplantation developed unilateral visual disturbance with a central scotoma. (3) Results: Ophthalmologic examination revealed unilateral maculopathy; a Tacrolimus macular toxicity was suspected. After drug discontinuation, a complete visual recovery was observed; however, the ultrastructural macular damage was irreversible. (4) Conclusions: Reports regarding maculopathy associated with Tacrolimus are limited. This case report adds to the current literature regarding the possible macular toxicity of this immunosuppressive agent, especially if it exceeds therapeutic serum levels. Further data are needed to confirm this possible association. A careful ophthalmologic examination should be promptly performed in patients manifesting visual disturbance while taking Tacrolimus to prevent irreversible, permanent vision loss due to possible drug toxicity.

## 1. Introduction

Tacrolimus, a fungal metabolite produced by Streptomyces tsukbaensis, is an immunosuppressive agent that inhibits calcineurin phosphatase, thereby irreversibly inhibiting T-cell activation, and the production of cytokines, which are normally activated in a strong immune response. It is most used in the setting of solid organ allogeneic transplants in the prevention of rejection or autoimmune diseases. Moreover, topical Tacrolimus eye drops are effective in the treatment of allergic ocular diseases and dry eye [1,2,3]. Topical Tacrolimus is generally well tolerated. The most common side effects include mild and transient ocular irritation and an increased risk of corneal infection [2]. Systemic Tacrolimus, however, can have serious yet seldom ophthalmic adverse effects, which have been reported in the literature to include cortical blindness [4,5] and optic neuropathy [6,7,8]. 

The mechanisms of tacrolimus-associated vision loss have not been fully elucidated. Several hypotheses exist. Tacrolimus may have a direct neurotoxic effect on the white matter due to its predilection for myelin because of its lipophilic nature, resulting in direct neurotoxicity leading to white matter lesions. Tacrolimus may also increase thromboxane A2 levels, thereby causing vasoconstriction and/or ischemia of the optic nerves. Tacrolimus-induced cortical blindness seems to have an acute onset and tends to be reversible after drug discontinuation [4]. The clinical course of tacrolimus-induced optic neuropathy can vary substantially, which can affect the degree of vision loss, laterality, ophthalmological findings, and visual recovery after Tacrolimus discontinuation [8]. Toxicity can occur at any Tacrolimus level and at any time after transplantation [6].

We describe a rare case of maculopathy as a possible complication of Tacrolimus therapy. The study followed the tenets of the Declaration of Helsinki. The patient provided informed consent for the research use of clinical records and data included in the study. Reports regarding maculopathy associated with the use of this immunosuppressive agent are limited [9].

## 2. Materials and Methods

A 56-year-old man was presented to our clinic with a history of sudden, painless diminution of vision in the left eye lasting 10 days. He reported seeing a “black spot” in front of his left eye, which obscured his clarity of vision. He denied any associated systemic or neurologic symptoms. He had a history of liver transplantation for alcoholic cirrhosis 10 months prior to presentation. He had been taking Tacrolimus (Advagraft, Astellas Pharma S.p.A.) at 10–12 mg/day. Therapeutic drug monitoring (TDM) was assessed every 2–4 days in the first month and then every 2 weeks. Tacrolimus dosage was adjusted according to TDM results to obtain therapeutic serum levels.

At the time of his visual symptoms, his Tacrolimus level was 8.20 ng/mL (therapeutic range 5–10 ng/mL). Two months before the onset of the visual symptoms, on the occasion of endoscopic retrograde cholangiopancreatography for extraction of the dislocated biliary prosthesis, Tacrolimus levels had elevated to 28.40 ng/ml. Renal and liver function tests were always within normal limits. The patient did not use any medication known to interact with Tacrolimus. For the first month after liver transplantation, the patient had also been taking Prednisone 25 mg/day, but he developed steroid-induced diabetes. Insulin therapy was thus started, and the steroid was discontinued. 

## 3. Results

On ophthalmic examination, the best-corrected visual acuity (BCVA) was 20/20 in the right eye and 20/32 in the left eye with +0.75 D correction in both eyes. Color vision was normal in both eyes. Eye movements and pupillary reflexes were normal. Slit-lamp examination of the anterior segment in both eyes was within normal limits. IOP by applanation tonometry was 12 mmHg in both eyes. A dilated fundus examination revealed normal discs and retinal vasculature with no signs of diabetic retinopathy in both eyes. A subtle alteration of macular reflex was noted ophthalmoscopically in the left eye.

**Figure 1:**
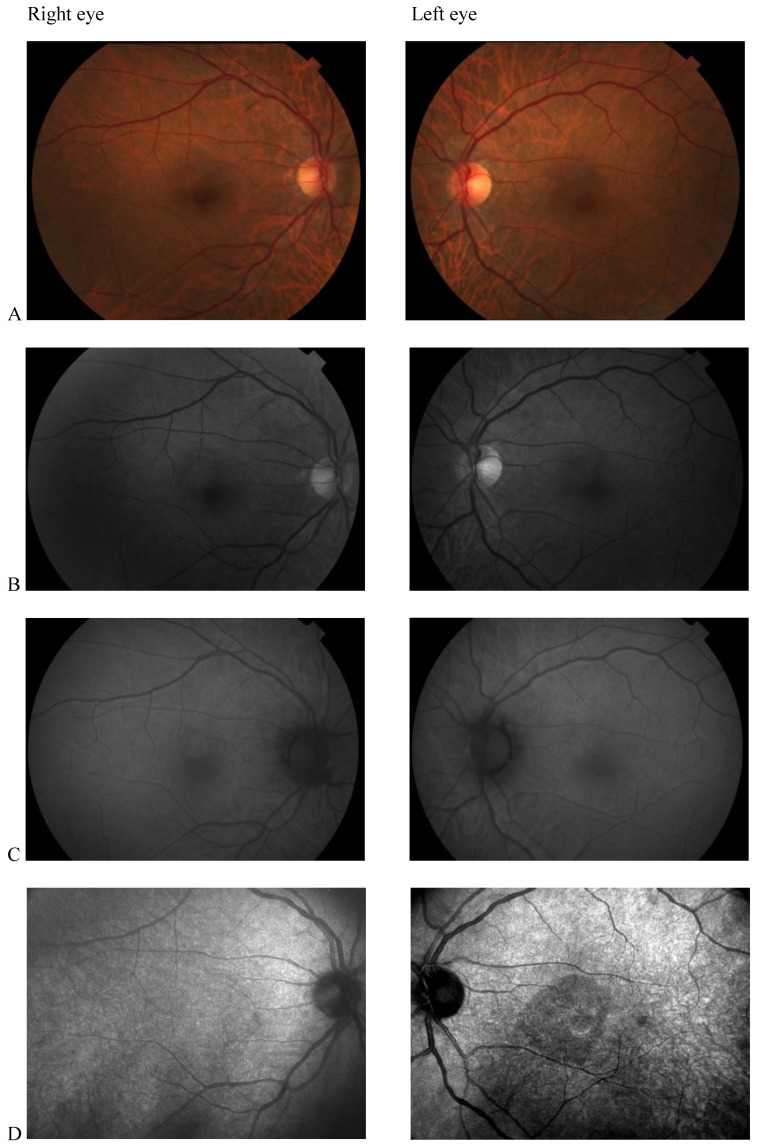
(**A**) Color fundus photography; (**B**) red-free fundus photography; (**C**) fundus autofluorescence; (**D**) Scanning laser ophthalmoscopy (SLO) fundus image revealed abnormal macular reflectivity in the left eye.

**Figure 2:**
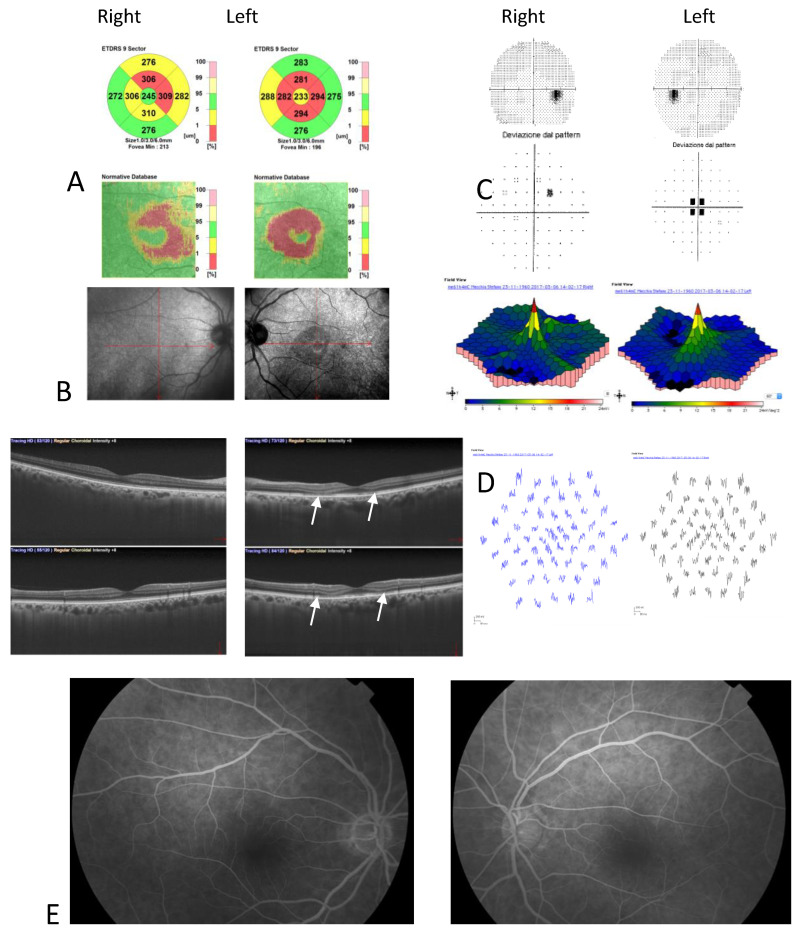
(**A**) Optical coherence tomography (OCT) Thickness Maps show a reduction of perifoveal macular thickness greater in the left eye; (**B**) OCT retinal segmentation analysis showed preserved retinal layers in the right eye and thinning of the outer nuclear layer in the left eye (arrows); (**C**) Humphrey’s visual field 30–2 total deviation map revealed central scotoma in the left eye; (**D**) Multifocal electroretinogram showed blunted foveal peak in the left eye. The voltage of the first positive wave (P1) was decreased in the temporal macula; (**E**) Fluorescein angiography was normal in both eyes.

Color fundus photography, red-free fundus photography, fundus autofluorescence, and fluorescein angiography were normal in both eyes. The abnormal macular reflex in the left eye was evident only on a scanning laser ophthalmoscopy (SLO) infrared fundus image obtained with optical coherence tomography (OCT) (OCT/SLO NidekRS-3000 ADVANCE) (Figure 1). OCT scans revealed a reduction of perifoveal macular thickness, which was greater in the left eye (Figure 2A). Retinal layers segmentation analysis showed a marked reduction of the outer nuclear layer (ONL) in the perifoveal region of the left eye (Figure 2B). Humphrey perimetric 30–2 threshold test showed a central scotoma in the left eye (Figure 2C). A multifocal electroretinogram showed blunted foveal peak in the left eye. The voltage of the first positive wave (P1) was decreased in the temporal macula (Figure 2D). Fluorescein angiography was normal in both eyes (Figure 2E). 

Based on these findings and after Pubmed research, a Tacrolimus-induced toxic maculopathy was suspected. Tacrolimus was discontinued in accordance with the hepatologist. Ciclosporin and mycophenolate mofetil were prescribed. The patient reported prompt resolution of visual disturbance, which supported us in our diagnostic hypothesis. The patient did not show laboratory or clinical signs of transplant rejection.

**Figure 3:**
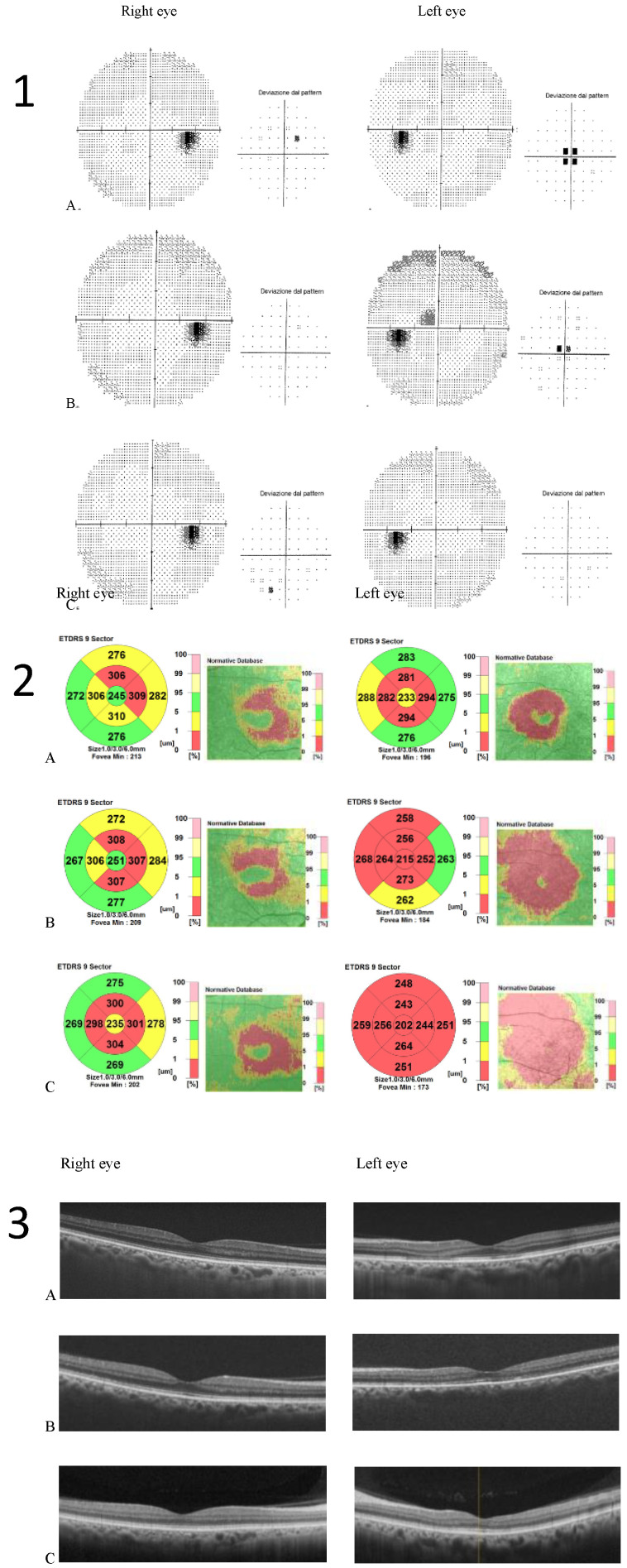
(**1**) Humphrey visual field 30–2 map and total deviation map (**A**). baseline; (**B**) 6 months after Tacrolimus discontinuation; (**C**) 3 years after Tacrolimus discontinuation. Progressive resolution of the left eye central scotoma is evident; (**2**) Optical coherence tomography (OCT) Thickness Maps (**A**) baseline, (**B**) 6 months after Tacrolimus discontinuation, (**C**) 3 years after Tacrolimus discontinuation. Macular thickness progressively reduced in the left eye more than in the right eye; (**3**) OCT retinal segmentation analysis of both eyes (**A**) baseline, (**B**) 6 months after Tacrolimus discontinuation, (**C**) 3 years after Tacrolimus discontinuation. The retina layers are preserved in the right eye and thinning of the outer nuclear layer is evident in the left eye.

After six months of Tacrolimus suspension, the left eye BCVA improved to 20/25, and the central scotoma detected by 30.2 perimetry was reduced. The patient did not show up at the one-year follow-up visit. He came back to our Clinic 3 years later, showing a complete resolution of visual disturbances. BCVA was 20/20 in both eye eyes, and visual field testing with Humphrey perimeter demonstrated complete resolution of left eye central scotoma (Figure 3(1)). Despite the functional recovery, however, OCT scans did not show anatomical recovery. Central retinal thickness progressively reduced in both eyes (Figure 3(2)). In his right eye, retinal thickness reduction involved only perifoveal sectors of the OCT macular map and was not associated with any alterations in retinal layers morphology. In his left eye, retinal thickness reduction was greater and involved all sectors of the OCT macular map. Retinal layer segmentation revealed severe thinning of ONL that remained stable over time (Figure 3(3)).

## 4. Discussion

Tacrolimus is an immunosuppressive agent that inhibits calcineurin phosphatase, thereby irreversibly inhibiting the development and function of T cells and the synthesis of cytokine. Three different formulations of Tacrolimus are available: the immediate release version (Prograft; Fujisawa), the slow-release version (Advagraf; Astellas), and the extended-release version (Envarsus; Veloxis). The principal advantages of the last two are the once-daily formulation and lower variation of serum levels [10]. Tacrolimus is primarily metabolized by cytochrome P4503A enzymes in the liver and intestinal mucosa and is excreted through the biliary route [11]. Metabolized products of Tacrolimus may still be biologically active; therefore, plasma concentrations of the drug may not reflect the total amount of active drug in the body [12].

Tacrolimus-induced optic neuropathy and cortical blindness have been reported in the literature; however, the exact mechanism is not fully understood. Induced direct neurotoxicity and vasoconstriction-induced ischemic damage have been proposed [6,7,8]. In our study, we describe a rare case of possible maculopathy associated with Tacrolimus therapy. To the best of our knowledge, Tacrolimus-induced maculopathy has only been reported in one other paper [9]. The case reported by Taehyuk et al. and our study were both based on mid-aged males that were using systemic therapy with Tacrolimus after liver transplantation. The patient in the previous case report had been taking Tacrolimus for 30 months and gradually developed bilateral visual disturbances. Unfortunately, the study did not report information regarding Tacrolimus plasma levels. 

Our patient experienced unilateral decreased vision after therapy with Tacrolimus for 10 months. Tacrolimus levels were in the normal range during the follow-up period; however, serum levels increased during routine checkups within close proximity of the onset of visual dysfunction. This elevation had occurred in association with ERCP and underlined the importance of careful dose adjustment in patients with conditions affecting the biliary system. Tacrolimus is excreted via the biliary system; thus, proper functioning of the biliary system could play a role in the severity of side effects in systemic medication. A similar elevation of Tacrolimus serum level several months before the onset of visual symptoms and normal drug level in proximity to the visual dysfunction has been reported in two cases of Tacrolimus-induced optic neuropathy [8]. This finding may raise the hypothesis that Tacrolimus accumulates in the tissues with consequent cumulative dose toxicity over time. Most of the Tacrolimus-induced optic neuropathy cases reported in the literature occurred despite normal levels of Tacrolimus. Although it is important to measure serum levels of Tacrolimus to ensure therapeutic levels, it is important to note that this may not be predictive of the development of ocular toxicity.

The clinical characteristics of these two cases regarding suspected Tacrolimus-associated maculopathy appeared significantly different, in that the previously reported patient did not show any structural alterations in the retinal architecture on the OCT scans. The angiography only showed a slight reduction of foveal reflex and a window defect caused by retinal pigment epithelium atrophy [9]. In our patient, the ocular fundus examination and the SLO images identified macular reflex alterations in the symptomatic left eye. OCT scans showed a bilateral reduction of central retinal thickness, which was more severe in the symptomatic left eye, in addition to significant thinning of left eye ONL. To the best of our knowledge, there are no other studies in the current literature that show an anatomically damaged retinal layer in Tacrolimus-associated maculopathy. This finding is consistent with the recent results of Casado et al. [13], that studied retinal damage in individuals with Hydroxychloroquine treatment. They found that ONL thickness was significantly decreased in patients taking Hydroxychloroquine compared with the control group. Authors suggested that this may be an early sign of Hydroxychloroquine retinal toxicity. The ONL contains the cell bodies of the photoreceptor cells. We hypothesize that, like Hydroxychloroquine, Tacrolimus may have a negative effect on the cell bodies of the photoreceptor. Although the exact mechanism is not known, detrimental factors could include a direct toxic effect or secondary damage due to induced vasoconstriction and/or ischemia.

The case reported by Taehuk et al. [9] and the study of Casado et al. [13] were key in our suspect diagnosis. The elevation of Tacrolimus plasma level in our patients directed us toward the hypothesis of Tacrolimus induced maculopathy. In accordance with the hepatologist that was managing the patient, Tacrolimus was quickly suspended. The immunosuppressive therapy was modified to include ciclosporin and mycophenolate mofetil. The prompt resolution of visual symptoms referred by the patient supported us in our diagnostic hypothesis. 

With regards to differential diagnosis, diabetic maculopathy needs to be considered. Vasoconstriction-induced macular damage or transient macular edema with subsequent retinal thinning or progressive retinal thickness reduction even in the absence of diabetic retinopathy cannot be excluded [14]. Previous pachychoroid pathologies in the left eye, such as central serous retinopathy during corticosteroid therapy, should also be considered. Previous vascular events to the left eye, such as transient arterial or venous occlusion, should also be included in the differential diagnosis. Factors related to a chronic unhealthy lifestyle related to excessive alcohol consumption and/or poor nutrition could play a role in inducing or worsening macular disease. 

## 5. Conclusions

Our case suggests the possibility of a Tacrolimus-induced maculopathy. This hypothesis is based on the evidence of damage to the macular ONL like that caused by other drugs with retinal toxicity such as Hydroxychloroquine and on the resolution of visual disturbance after discontinuation of the drug. Macula damage induced by other causes, however, cannot be excluded and need to be carefully assessed in the differential diagnosis. 

Although rarely reported in the literature, clinicians should be aware of the potential ocular toxicity of Tacrolimus. Prompt ophthalmologic examination with OCT scans and visual field examination should be performed in cases of visual disturbance in patients taking this immunosuppressive agent, especially if Tacrolimus plasma levels exceed therapeutic ranges. Considering the lack of standardized protocols and the rarity of the clinical manifestations, OCT scans, visual fields, and other diagnostic examinations should be repeated periodically based on the severity of symptoms and should be managed on a case-to-case basis. Immediate identification of Tacrolimus ocular toxicity and consequent drug discontinuation is of utmost importance to prevent severe and permanent vision loss.
